# A Neural Network Model Combining [-2]proPSA, freePSA, Total PSA, Cathepsin D, and Thrombospondin-1 Showed Increased Accuracy in the Identification of Clinically Significant Prostate Cancer

**DOI:** 10.3390/cancers15051355

**Published:** 2023-02-21

**Authors:** Francesco Gentile, Evelina La Civita, Bartolomeo Della Ventura, Matteo Ferro, Dario Bruzzese, Felice Crocetto, Pierre Tennstedt, Thomas Steuber, Raffaele Velotta, Daniela Terracciano

**Affiliations:** 1 Nanotechnology Research Centre, Department of Experimental and Clinical Medicine, University Magna Graecia of Catanzaro, 88100 Catanzaro, Italy; 2 ElicaDea, Spinoff of Federico II University, 80131 Naples, Italy; 3 Department of Translational Medical Sciences, University of Naples “Federico II”, 80131 Naples, Italy; 4 Department of Physics “Ettore Pancini”, University of Naples “Federico II”, 80126 Naples, Italy; 5 Division of Urology, European Institute of Oncology (IEO), IRCCS, 20141 Milan, Italy; 6 Department of Public Health, Federico II University of Naples, 80131 Naples, Italy; 7 Department of Neurosciences, Sciences of Reproduction and Odontostomatology, University of Naples Federico II, 80131 Naples, Italy; 8 Martini-Klinik, University Hospital Hamburg-Eppendorf, 20246 Hamburg, Germany

**Keywords:** artificial neural network, Phi, PCLX, prostate cancer, tumor markers

## Abstract

**Simple Summary:**

The widespread use of PSA for prostate cancer diagnosis significantly contributed to the high rate of overdiagnosis and overtreatment. For several years, the Prostate Health Index (PHI) has been proposed as a tool able to improve PSA specificity. More recently, the Proclarix test has been developed. The combination of these two tests promises to ameliorate risk stratification of PCa patients at initial diagnosis. In this study, we evaluated the performance of an artificial-neural-network-based model combining kallikrein markers included in PHI and the cancer-related markers of Proclarix for the prediction of positive biopsy and high-grade cancers. Our findings suggested that the combined model had an increased accuracy in the identification of pathological aggressive PCa at initial diagnosis.

**Abstract:**

Background: The Prostate Health Index (PHI) and Proclarix (PCLX) have been proposed as blood-based tests for prostate cancer (PCa). In this study, we evaluated the feasibility of an artificial neural network (ANN)-based approach to develop a combinatorial model including PHI and PCLX biomarkers to recognize clinically significant PCa (csPCa) at initial diagnosis. Methods: To this aim, we prospectively enrolled 344 men from two different centres. All patients underwent radical prostatectomy (RP). All men had a prostate-specific antigen (PSA) between 2 and 10 ng/mL. We used an artificial neural network to develop models that can identify csPCa efficiently. As inputs, the model uses [-2]proPSA, freePSA, total PSA, cathepsin D, thrombospondin, and age. Results: The output of the model is an estimate of the presence of a low or high Gleason score PCa defined at RP. After training on a dataset of up to 220 samples and optimization of the variables, the model achieved values as high as 78% for sensitivity and 62% for specificity for all-cancer detection compared with those of PHI and PCLX alone. For csPCa detection, the model showed 66% (95% CI 66–68%) for sensitivity and 68% (95% CI 66–68%) for specificity. These values were significantly different compared with those of PHI (*p* < 0.0001 and 0.0001, respectively) and PCLX (*p* = 0.0003 and 0.0006, respectively) alone. Conclusions: Our preliminary study suggests that combining PHI and PCLX biomarkers may help to estimate, with higher accuracy, the presence of csPCa at initial diagnosis, allowing a personalized treatment approach. Further studies training the model on larger datasets are strongly encouraged to support the efficiency of this approach.

## 1. Introduction

Widespread use of prostate specific antigen (PSA) for the early detection of prostate cancer (PCa) led to a high number of unnecessary biopsies and increased diagnosis of indolent PCa [1]. This low specificity prompted the researchers to find new biomarkers not only for early PCa detection, but also for better discrimination of clinically significant PCa (csPCa, defined as GG ≥ 2) [2]. In this context, several new tests have been proposed—one of the most promising is the prostate health index (PHI; Beckman Coulter). The PHI score is calculated combining the values of PSA, %fPSA, and [-2]proPSA. Several authors showed that PHI has a good ability to detect csPCa [3]. More recently, the Proclarix (Proteomedix, Switzerland) blood test has been developed. The Proclarix score is obtained using thrombospondin-1 (THBS1), cathepsin D (CTSD), total PSA (tPSA), free PSA (fPSA), and patient age values [4]. We previously demonstrated that the combination of PHI and Proclarix test results synergistically improved the diagnostic performance of the individual test [5]. On this basis, we hypothesized that it could be valuable to explore whether a new model comprising the kallikrein markers included in PHI and the cancer-related markers of Proclarix may achieve higher values of sensitivity and specificity for the detection of both all cancers and csPCa than the tests alone. To address this issue, considering the complexity of the problem, we used an artificial neural network (ANN)-based approach. The number of potential variations that can be derived from a combination of five variables extracted by different subjects is very large. ANN is better than deterministic models to describe such extremely complex datasets and to reveal laws of the relationships among variables by analysing the output of the system [6,7]. We developed a deep learning algorithm that can identify PCa and csPCa from a panel of five clinical variables plus the patient’s age. The levels of (i) total PSA (tPSA), (ii) free PSA (fPSA), (iii) the isoform-2 of proPSA (p2PSA), (iv) thrombospondin-1 (THBS1), and (v) cathepsin D (CTSD) were determined in 344 patients using automated immunometric and ELISA assays. Based on our previous studies [1,2,3,4,5,8,9,10], a model containing those variables can potentially detect PCa and csPCa early with higher accuracy compared with PHI and Proclarix individual test results. As shown in a recently published systematic review [11], several studies demonstrated the potential of ANNs for the identification and validation of PCa biomarkers. PSA and the other biomarkers are commonly used for PCa diagnosis, prognosis, and follow-up. However, an AI-based approach can provide several advantages such as managing enormous data sets, identifying complex relationships, and using very few resources. Our data confirmed that such an approach is useful to identify new tools for clinical management of PCa. Moreover, according to other authors [12,13], we found that the ANN model has the highest accuracy to predict all cancers and csPCa.

## 2. Materials and Methods

### 2.1. Study Population

Serum samples (*n* = 344) were collected at two clinical centres: 159 (97 with PCa) from the Department of Translational Medical Sciences, Federico II University, Naples, Italy, and 185 (91 with PCa) from the Martini-Klinik, University Hospital Hamburg-Eppendorf, Hamburg, Germany. All samples were obtained consecutively from May 2020 to July 2021 (Naples) and from 2013 to 2016 (Hamburg) before prostate biopsies. A systematic 12-core TRUS-guided biopsy was executed in Hamburg, while patients in Naples had mpMRI-guided biopsy. Primary and secondary Gleason scores (GS) were assigned by a genitourinary pathologist, according to the 2005 consensus conference of the International Society of Urological Pathology definitions [14]. Men with total PSA values between 2 and 10 ng/mL were included and subjects receiving drugs (i.e., 5-alpha reductase inhibitors) were excluded from the study. Prostate volume was assessed by TRUS. The study was approved by the local ethics committees (prot. n. 118/20 for the institutional Ethics Committee of the University of Naples Federico II). Participants provided written approved consent.

### 2.2. Determination of Proclarix and PHI

Patients had blood drawn before digital rectal exploration (DRE). Whole blood was allowed to clot before serum was separated by centrifugation. Serum aliquots were stored at −80 °C until samples were processed, according to Semjonow et al. [15]. PSA, fPSA, and p2PSA were measured by Access2 Immunoassay System analyzer (Beckman Coulter, Brea, CA, USA) calibrated against the WHO standard for PSA and fPSA. The analytical performance of the measurements assessed with control materials (Beckman Coulter) showed values within the allowed recommended limits. Measurement of Proclarix was performed by CTSD and THBS1 ELISA from the Proclarix kit (Proteomedix), as described previously [4]. Total PSA and free PSA values were determined using a Cobas system (Roche) for Hamburg samples and on an Access2 Immunoassay System analyzer (Beckman-Coulter) calibrated against the WHO standard for Naples samples. In both cases, the Proclarix score was calculated according to the instruction for use using the online risk-score calculator (www.proclarix.com/risk/calculator, accessed on 13 October 2021). PHI measurements were conducted at University Federico II (Naples, Italy) for all samples, according to the manufacturer’s instructions for use.

### 2.3. Neural Networks Design

Clinical and non-clinical variables of patients suspected of having PCa were processed by a deep neural network model. We used as input variables the values of total PSA, free PSA, p2 PSA, CTSD, and THBS1, determined following the procedure described above. In some tests and more sophisticated versions of the model, we used the age of the patients as an additional input variable. The deep neural network model that we have used in this work is composed of an input layer, an output layer, and four other layers in between. These extra intermediate layers are the functions that operate sequentially on the data, transforming an originating set of variables (input) into a diagnosis on cancer (output), and representing the kernel of the algorithm. In this study, after testing different combinations of neurons and layers, we found that the sequence of layers that optimizes performance is as follows: (1) linear layer, (2) batch normalization layer, (3) non-linear (hyperbolic tangent function) layer, and (4) linear layer. The output layer is represented by a 0/1 data type variable B, such that, if B = 1 (0), the patient is diagnosed (is not diagnosed) with cancer by the model template. The number of nodes in the input layer is 5 (6 when considering age), onto which values of the variables are passed. The number of nodes of the intermediate layers is 6 (7 when considering age). The neural network code was written in Mathematica. Layers of the network were concatenated using the function NetGraph(), then the function NetInitialize() was used to instantiate the neural network model. The algorithm was trained using datasets of varying sample size; elements in the training datasets were chosen randomly from an initial database (O) of 344 patients. For each training set (T), the corresponding validation (V) set was determined as the difference between two sets O and T: V = O − T. The values of total PSA, free PSA, p2 PSA, CTSD, and THBS1, plus age, were concatenated to create a single feature set conveyed to the model input. The training procedure was accomplished using an epoch number of 8000 samples and a batch size of 1024 elements. To test performance, for each neural network configuration, training, and validation sample size, we performed 100 different simulations. As elements in the training and validation sets were selected randomly, in each of these model runs, the composition of the sample was different from the others, causing, as a result, a different output of the neural network model under the same values of parameters and model conditions. In all cases, the neural network model achieved convergence and the error loss settled to within an error range of less than 1% of the steady state value.

### 2.4. Training and Validation of the Neural Network Model

We used training datasets with variable sizes during the learning process to fit the model parameters of the classifier. For the classification task, we used supervised machine learning, i.e., we used labelled datasets to train the algorithm in a way that it may make the diagnosis of PCa accurate. Training samples were labelled using the GS of the patient, i.e., an external variable determined independently from the values of total PSA, free PSA, p2 PSA, CTSD, THBS1, and age, used as input in the neural network model. The GS is an external estimate of the state of a patient and is considered here as the ground truth diagnosis on cancer. The GS is a set of two numbers, the combination of which describes the severity of PCa. The higher the GS, the more likely that the cancer will grow and spread quickly. Cases with no reported GS are considered not clinically relevant. Here, we used two different training criteria based on the GS of data: (i) training without GS stratification, where we categorize samples as cancer-positive if they have GS, however high; and (ii) training with GS stratification, in which case we assumed that samples with a value of GS < 7 (≥7) are indolent (cancer). Parameters and weights of the neural network model were then tuned to match the GS prediction on cancer. To assess the performance of the model, we used validation or test datasets independent of the training data sets, i.e., never used during the training procedure. In doing so, we minimized overfitting. The model’s performance was measured using sensitivity and specificity. The sensitivity (specificity) of a binary classifier is the proportion of positive (negative) results that are true positives (negative).

## 3. Results

### The ANN Model Performance

Our results showed how the neural network (NN) model with the five variables of total PSA, free PSA, p2 PSA, CTSD, and THBS1, plus age, compares to simpler linear regression models of the sole PHI (LRPhi) or Proclarix variable (LRPro). The NN model was built to receive as input the clinical variables associated with patients suspected to have prostate cancer, and to generate as an output the diagnosis of cancer. To train and validate the model, predictions of the NN algorithm were compared to the true health status of the individuals determined through the conventional Gleason score grading system, as described in the methods. Models were trained on a dataset with a size varying from 130 to 260 elements and were validated on a complementary dataset of 215 to 85 elements, such that the original sample size is 344.

**Figure 1:**
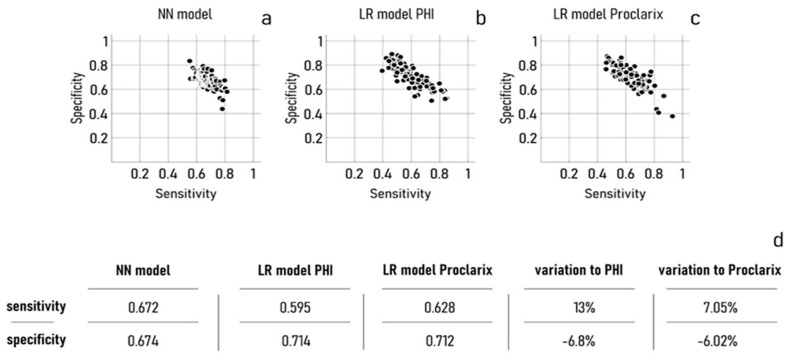
Performance of the neural network model in terms of sensitivity and specificity (**a**), compared to a linear logistic regression model of the PHI (**b**) and Proclarix variable (**c**). In these tests, the neural network model was built to receive, as an input, the pool of five variables: total PSA, free PSA, p2 PSA, CTSD, and THBS1, plus age. In determining performance, samples were stratified using information on the Gleason score. Patients with a value of GS < 7 were considered healthy. Mean values of sensitivity and specificity for the NN and LR models, evaluated over 100 simulations (**d**).

Here, we present the results of an NN model of all clinical variables plus age. Moreover, in determining whether patients with known levels of total PSA, free PSA, p2 PSA, CTSD, and THBS1—and known age—have csPCa, we used information on the GS. Individuals with a value of GS ≥ 7 are diagnosed with csPCa. Individuals without or with GS < 7 are not. We measured the performance of the model using the sensitivity (true positive rate) and specificity (true negative rate) of the classification of the model, trained on a dataset of 180 elements, and evaluated on a validation dataset of 164 elements. Moreover, to improve the accuracy and statistical significance, we evaluated the model’s performance against 100 different randomized training and validation datasets, sampled from an original population of 344 elements (Figure 1a). The efficiency of the NN model was compared to the efficiency of a simple linear logistic regression (LR) model of the PHI (Figure 1b) and Proclarix (Figure 1c) variable.

The average values of sensitivity (se) and specificity (sp) determined for the models are se = 0.672 and sp = 0.674 for the NN model, se = 0.595 and sp = 0.714 for the LRPhi model, and se = 0.628 and sp = 0.712 for the LRPro model, respectively (Figure 1d).

The results of the classification test indicate that the NN model provides an improvement over the LR model of PHI and Proclarix in terms of sensitivity, with a measured enhancement of ~13% with respect to PHI (*p* < 0.0001) and ~7% with respect to Proclarix (*p* = 0.0001). The results also indicate that the specificity of the classification of the NN model is lower than the specificity measured for the LR model of PHI (*p* = 0.0003) and Proclarix (*p* = 0.0006), with a decrease of ~6.8% and ~6%, respectively (Figure 1d).

**Figure 2:**
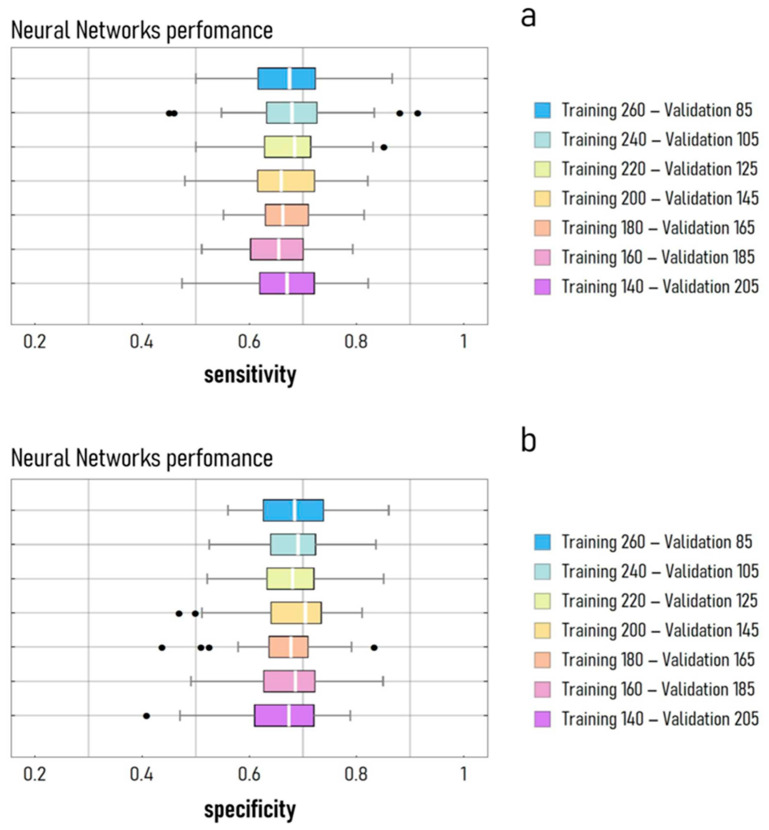
Box and whisker plot of the sensitivity (**a**) and specificity (**b**) of the NN model in performing the diagnosis of patients with prostate cancer. In determining the values of sensitivity and specificity, we used information on the tumour Gleason score determined at RP. Patients with a value of GS < 7 were considered healthy. In tests, the neural network model was built to receive, as an input, the pool of five variables: total PSA, free PSA, p2 PSA, CTSD, and THBS1, plus age. Descriptive statistics plots were determined as a function of the training and validation sample size. The left and right boundary, in each box plot, corresponds to the 25% and 75% quartiles, respectively, of the distributions, while the thick white band marks the median value. Black points are the distribution outliers.

An extended test campaign aimed at measuring the performance of the NN model as a function of sample size indicated that the values of sensitivity and specificity of the model showed moderate sensitivity to the number of elements in the training and validation set (Figure 2). For values of the training (validation) dataset dimension varying between 140 (205) and 260 (85), we found that the mean values of sensitivity and specificity of the model oscillated between 0.65 and 0.68 and 0.66 and 0.69, respectively. For each considered size, the distributions of sensitivity and specificity determined for 100 different randomized models are described by the box and whisker plots in Figure 2a (sensitivity) and Figure 2b (specificity).

The results shown in Figure 1 and Figure 2 are relative to a model of all considered clinical variables plus age, which makes use of the complete consolidated knowledge on the Gleason score (model with GS stratification). However, to examine whether the model results are affected by the model design, database composition, and rules of inference, we performed several additional tests in which the working parameters of the tests were varied. Using different model templates, different values of the model parameters, and different combinations of the input variables, we generated many output results. Below, we report the results in the same diagrams to enable a direct comparison between models and to draw general conclusions.

**Figure 3:**
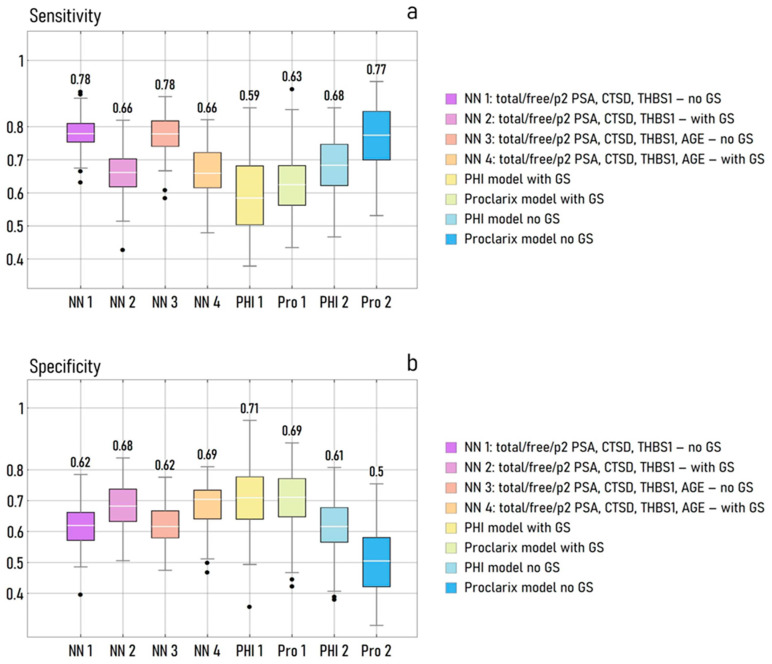
Descriptive statistics plots illustrating the sensitivity (**a**) and specificity (**b**) of different neural networks and logistic regression models. The models were designed to receive as an input the levels of total/free/p2 PSA, the levels of CTSD and THBS1, and the age of patients (NN models); the PHI and Proclarix test (LR model). Moreover, models were conceived to either use or not information about the GS of the patients. Differences between model designs and conditions are reflected by different values of sensitivity and specificity of the classification of tumour cases in 140-element validation datasets.

Figure 3a shows the box and whisker plots of the values of sensitivity, determined over 100 different tests as a function of the model design. The results were determined for the NN of the input variables total PSA, free PSA, p2 PSA, CTSD, and THBS1, with (NN2) or without (NN1) GS stratification, and for the neural networks of the same variables plus age, with (NN4) or without (NN3) GS stratification. These values of sensitivity were compared to those found for the linear logistic regression (LR) model of the PHI variable, with (PHI1) and without (PHI2) GS stratification, and to the values of sensitivity relative to a LR model of Proclarix, with (PRO1) and without (PRO2) GS stratification. The neural network model of the total PSA, free PSA, p2 PSA, CTSD, and THBS1 performed better than the others, with a mean value of sensitivity se ~0.78, either considering or not the additional variable age. In contrast, the sensitivity associated with the Proclarix variable was se ~0.77. The sensitivity of all other model templates was lower than se ~0.70.

Figure 3b shows the box and whisker plots of the values of specificity, determined for the same model templates and training and validation datasets, used for determining sensitivity and described above. In contrast to sensitivity, the higher value of specificity was achieved by the LR model of PHI with no GS stratification (spe ~0.71), followed by the NN model of the complete set of variables and by the LR model of Proclarix, both with GS stratification (spe ~0.69). All other values of specificity ranged between 0.61 and 0.68. The results presented in this section are relative to a training (validation) sample size of 200 (145) elements.

**Figure 4:**
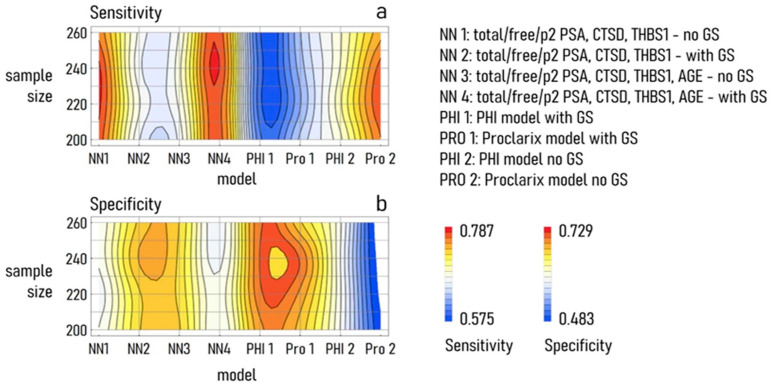
Density plots of sensitivity (**a**) and specificity (**b**) of different neural networks and logistic regression models as a function of the model design and size of the training and validation set.

To verify whether performance was mainly due to the model design, we determined the values of sensitivity and specificity of the classification as a function of the model design and of the sample size, changed between 200 and 260. Density plots of the resulting values of sensitivity (Figure 4a) and specificity (Figure 4b) demonstrated that the model’s performance showed a very high sensitivity to the model characteristics, and was only moderately influenced by sample size, at least for the range of dimensions considered in this study. Thus, the NN model without GS stratification achieved higher sensitivity than the Proclarix test and significantly higher sensitivity than the PHI test. Regarding specificity, there was not a model that performs significantly better than the others.

The results presented in Figure 3 and Figure 4 also indicated that the variability of the NN model output was lower than the variability of the PHI or Proclarix test. As low levels of variation were associated with high accuracy, high precision, and elevated repeatability, collectively, the results suggested that the NN model of total, free PSA, and p2 PSA, as well as of CTSD and THBS1, may be a valuable tool to assess the diagnosis of PCa.

## 4. Discussion

Overdiagnosis and overtreatment of indolent PCa are well-known consequences of PSA use [16,17]. Several new urine- and blood-based tests as well as risk calculators showed promising results [2,18], but none are available in routine clinical practice. Thus, there is an urgent clinical need for new tools able to ensure, at the same time, early detection of PCa and no overtreatment.

Men with low-risk cancer at initial diagnosis have the option of active surveillance (AS) and deferred treatment, avoiding invasive treatment (radical prostatectomy or radiation therapy) and the associated harmful side effects (81% patients develop erectile dysfunction, 17% urinary incontinence, and 12% bowel dysfunction) [19].

At present, the D’Amico/NCCN and EAU risk stratification systems or Partin’s table, developed more than a decade ago, are the gold standard to select patients for AS. More recently, several new biomarkers have been associated with csPCa [2], and the use of a non-invasive test to stratify the risk of aggressive PCa is an appealing strategy.

In this study, we described the ability of an ANN-based model including total PSA, free PSA, p2 PSA, CTSD, and THBS1 to detect high-grade PCa in an initial biopsy cohort of men aged ≥50 years.

We selected the blood-based proteins of PHI and Proclarix tests as several authors showed that they were significantly associated with an increased risk for a positive biopsy and for a GS of ≥7 at RP [5,20,21,22].

Applying a deep learning strategy, we demonstrated that the addition of CTSD and THBS1 to the three different PSA molecular forms included in PHI calculation showed the highest accuracy and precision for the identification of all cancers and high-grade PCa (GS ≥ 7).

The sensitivity and specificity obtained for the ANN model including age demonstrated comparable values to the model when age was excluded, suggesting that circulating biomarkers could be much stronger predictors than age. Accordingly, several new blood-based tests have been proposed in the last decades as tools able to identify csPCa at initial diagnosis [23].

Combining the single biomarkers of PHI and Proclarix by the ANN approach rather than the final scores by logistic regression analysis, as in our previous work [5], we achieved an improvement in specificity (68% vs. 49%, respectively) for csPCa identification.

We previously applied ANNs to predict csPCa, combining only PSA molecular forms with PSA density [9] or PHI with mpMRI [10], showing encouraging results for this approach. A few other authors used this approach to explore the potential of circulating biomarkers to predict PCa grading [12] and of ANN models to select men for biopsy [24,25].

Computational intelligence models are near to ideal as tools to identify new strategies for PCa diagnosis and prognosis, as they can deal with the indeterminateness of biomarkers values. It is well recognized that, in every single patient, it is likely one will find different abnormal biomarker results, and their combination is a better way to assess PCa diagnosis and prognosis [26].

For an AI tool to be clinically useful, two conditions must be addressed. First, it must provide the clinicians with information not available via the conventional clinical tools. Second, the tool must provide information affecting patients’ clinical management. On this basis, there are several areas of potential clinical utility in the case of prostate cancer: (i) prediction of biopsy outcomes (benign prostatic hyperplasia vs. cancer); (ii) prediction of PCa grading at RP (low-grade vs. high-grade); (iii) prediction of response to conventional therapy (responders vs. non-responders); and (iv) prediction of PCa relapse after surgery (relapsers vs. non-relapsers).

In all of these cases, it is not possible to assess the clinical outcome based on the initial data, but such an assessment is able to affect clinical management. So, the clinical utility of such a tool is not necessarily assured by high levels of sensitivity and specificity. Accuracy, sensitivity, and specificity do not necessarily need to be very high to impact clinical management [27]. For example, when the clinical decision may evolve during the disease, even a mild improvement in prediction (e.g., from 50 to 70%) may be considered clinically useful. In addition, tools with the potential to impact the patient’s perceptions and behaviors about their disease could also have some clinical utility.

The results produced by our study will also provide researchers with data to bridge the gap between models and tools. If tested on a large amount of clinical data at multiple centers, clinical utility will be reinforced. To overcome the obstacles in the clinical translation of the AI model, robust clinical evaluation, via easy-to-use measurements of quality of care and patient outcomes, will be essential. Further investigation is required (1) to identify biases of algorithms and develop actions to address these, (2) to improve generalizability, and (3) to create methods able to facilitate the interpretability of machine learning results. If these goals will be achieved, the clinical utility for patients is likely to be guaranteed.

In addition, ANN models showed performance comparable to logistic regression models in predicting csPCa [28], and such an approach associated with sensors [29] could represent an innovative method for large-scale, rapid, and low-cost PCa screening, aiming to avoid overdiagnosis and overtreatment.

Our study has some limitations. Firstly, our study includes data of two populations from two different clinical centres, thus we have no centralized pathology evaluation. Secondly, it includes only patients with tPSA between 2 and 10 ng/mL and we did not evaluate how the model performs in patients with tPSA values higher than 10 ng/mL, who are at higher risk of PCa.

However, our present study highlighted the high potential of ANN models for the prediction of PCa aggressiveness. The proposed model combining PHI and Proclarix biomarkers, once prospectively validated on a large study population, could represent an easy-to-use and widely accessible tool to distinguish, at initial diagnosis, patients who harbour high-grade PCa and really need invasive treatment. Thus, it might be useful for a personalized clinical decision-making process. Despite that the combination of five biomarkers is expensive as a first-line test, it will lead to a reduction in the costs of the entire diagnostic-therapeutic pathway of PCa patients, in terms of biopsies, surgical interventions, and the avoidance of drugs for post-surgical complications. Large prospective validation is needed to assess whether our model is associated with overall survival, PCa-specific mortality, and progression-free survival.

## 5. Conclusions

In conclusion, an ANN model including PSA, fPSA, p2PSA, CTSD, and THBS1 showed improved accuracy for the detection of high-grade cancer. Such a model may provide information on disease aggressiveness at initial diagnosis by a non-invasive method, favoring a personalized treatment choice. Thus, these findings strongly encourage to explore the use of ANN-based approaches to identify new tools for the management of PCa patients.

## Data Availability

The data that support the findings of this study are available upon request from the corresponding authors (F.G. and D.T.).
